# Monocyte Expression and Soluble Levels of the Haemoglobin Receptor (CD163/sCD163) and the Mannose Receptor (MR/sMR) in Septic and Critically Ill Non-Septic ICU Patients

**DOI:** 10.1371/journal.pone.0092331

**Published:** 2014-03-17

**Authors:** Anders G. Kjærgaard, Sidsel Rødgaard-Hansen, Anders Dige, Jan Krog, Holger J. Møller, Else Tønnesen

**Affiliations:** 1 Aarhus University Hospital, Department of Anaesthesiology and Intensive Care, Aarhus C, Denmark; 2 Randers Regional Hospital, Department of Anaesthesiology and Intensive Care, Randers, Denmark; 3 Aarhus University Hospital, Department of Medicine V (Hepatology and Gastroenterology), Gastro-Immuno Research Laboratory (GIRL), Aarhus C, Denmark; 4 Aarhus University Hospital, Department of Clinical Biochemistry, Aarhus C, Denmark; St. Joseph's Hospital and Medical Center, United States of America

## Abstract

**Background:**

The diagnosis of sepsis is challenging and there is an unmet need for sensitive and specific diagnostic and prognostic biomarkers. Following activation of macrophages and monocytes, the haptoglobin-haemoglobin receptor (CD163) and the mannose receptor (MR) are shed into the circulation (sCD163 and sMR).

**Objective:**

We investigated monocyte expression of CD163 and MR, and levels of sCD163 and sMR in septic and non-septic patients, and in healthy controls. We hypothesised that these receptors are elevated during sepsis and can be used diagnostic and prognostic.

**Methods:**

Twenty-one patients with severe sepsis or septic shock and 15 critically ill non-septic patients were included in this prospective observational study at three ICUs at Aarhus University Hospital and Randers Regional Hospital, Denmark. Fifteen age- and gender-matched healthy volunteers served as controls. Levels of sCD163 and sMR were measured using a sandwich ELISA and monocyte expression of CD163 and MR was evaluated by flow cytometry during the first four days of ICU stay. The diagnostic and prognostic values of the receptors were assessed using AUROC curves.

**Results:**

At ICU admission and during the observation period, monocyte expression of CD163 and levels of sCD163 and sMR were significantly higher in septic patients compared with non-septic patients and healthy controls (*p*<0.01 for all comparisons). Monocytes did not express MR. The diagnostic values estimated by AUROC were 1.00 for sMR, 0.95 for sCD163, 0.87 for CRP, and 0.75 for monocyte-bound CD163. Among the septic patients, monocyte expression of CD163 was higher in non-survivors compared with survivors at ICU admission (*p* = 0.02) and during the observation period (*p* = 0.006). The prognostic value of monocyte-bound CD163 estimated by AUROC at ICU admission was 0.82.

**Conclusion:**

The macrophage-specific markers CD163, sCD163, and sMR are increased in septic patients. Particularly sMR is a promising new biomarker of sepsis.

## Introduction

The diagnosis and management of sepsis poses a substantial challenge in the treatment of critically ill patients. As a result, there is an unmet need for sensitive and specific diagnostic and prognostic markers to predict disease severity and outcome [1]. Macrophages play a key role in sepsis and specific monocyte and macrophage-derived serum markers are promising markers in sepsis [2], [3].

The haptoglobin-haemoglobin receptor (CD163) and the mannose receptor (MR (CD206)) are scavenger receptors that are highly restricted to monocytes/macrophages and dendritic cells. The monocyte/macrophage surface expression of both CD163 and CD206 are increased in sepsis, and are regarded markers of monocyte/macrophage activation [4]. A soluble form of the CD163 receptor (sCD163) is produced by proteolytic cleavage of the extracellular domain and is found in serum from healthy individuals [5]. The shedding of CD163 is a constitutive process, but it can be increased by various stimuli, such as lipopolysaccharide (LPS) [6], [7]. Increased serum concentrations of sCD163 have been reported in a number of inflammatory diseases [3], including liver disease [8], diabetes [9] and infection [10]. We recently demonstrated that there also exist a soluble form of the mannose receptor (sMR) in human serum, and the highest values were detected in intensive care unit (ICU) patients [11]. The mannose receptor has a broad range of ligands, and its functions include degradation of endogenous glycoproteins and endocytosis of microorganisms [12]. It was previously reported that critically ill patients have increased levels of sCD163 compared with healthy controls [13]. Furthermore, the levels of sCD163 has been found superior to both C-reactive protein (CRP) and procalcitonin in differentiating between septic and non-septic patients [14].

In this study, we investigated whether macrophage-related biomarkers that reflect infectious inflammation in sepsis could be used diagnostic and prognostic and whether they are superior to the non-specific CRP [15].

We hypothesised that serum concentrations of sCD163 and sMR and the expression of monocyte-bound CD163 and MR are higher in septic patients compared with critically ill non-septic patients and healthy controls and that they can predict mortality within the group of septic patients. Our aim was to investigate whether these potential biomarkers could be used diagnostic to discriminate between septic and critically ill non-septic patients and prognostic to discriminate between survivors and non-survivors in an ICU setting. To elucidate the potential dynamic changes in biomarkers during the course of sepsis we followed patients during the first four days of their ICU stay.

## Materials and Methods

### Study design & patients

This prospective observational study was approved by The Central Denmark Region Committees on Health Research Ethics (reg. no. M-20080124) and the Danish data protection agency (reg. no. 2008-41-2421). The study was performed in accordance with the principles in the Helsinki Declaration. This study included 36 ICU patients at three different ICUs at Aarhus University Hospital and Randers Regional Hospital, Denmark. Written informed consent was obtained from the subjects if possible. If the patient was unable to give informed consent, this was obtained from the closest relative and the patients’ general practitioner. Twenty-one patients with severe sepsis or septic shock according to the criteria given by Bone and colleagues [16] and fifteen critically ill non-septic patients were included in the study (Table 1). All non-septic patients fulfilled the systemic inflammatory response syndrome (SIRS) criteria and had organ dysfunction in combination with an acute physiology and chronic health evaluation II (APACHE II) score above 13 at ICU admission. Fifteen healthy age- and gender-matched volunteers served as controls.

**Table 1:** Patient demographics.

			Sepsis	Non-sepsis	Control
			*n* = 21	*n* = 15	*n* = 15
**Demographics**					
Age, median (IQR)			66 (62−79)	58 (47−68)	61 (59−63)
Female gender, n (%)			10 (48)	8 (53)	9 (60)
**Cause of admission**					
Medical			13		
Surgical			8		
Trauma				4	
Intracranial haemorrhage				11	
**Severity of disease, median (IQR)**					
APACHE II score			17 (14−20)	18 (15−23)	-
SOFA score			8 (7−12)	8 (6−11)	-
**Treatment, n (%)**					
Respirator/NIV			18 (86)	11 (73)	0 (0)
Glucocorticoids			13 (62)	0 (0)	0 (0)
Dialysis			2 (10)	0 (0)	0 (0)
Inotropic agents	None		5 (24)	5 (33)	15 (100)
	1 agent		8(38)	10 (67)	0 (0)
	> 1 agent		7 (33)	0 (0)	0 (0)
	> 2 agents		1 (5)	0 (0)	0 (0)
Antibiotics	None		0 (0)	10 (67)	15(100)
	monotherapy		3 (14)	3 (20)	0 (0)
	polytherapy		18 (86)	2 (13)	0 (0)

APACHE II Acute Physiology And Chronic Health Evaluation II, SOFA Sequential Organ Failure Assessment, NIV Non-invasive ventilation.

We intended to include 15 patients in each group. Due to equipment failure, serum samples, but not peripheral blood mononuclear cells (PBMC), from six septic patients were lost. To reach the intended 15 patients in the septic group, additionally six septic patients were included. Subsequently n = 15 when reporting results on sCD163 and sMR, and n = 21 when reporting results on monocyte expression of CD163 and MR in the septic group.

The exclusion criteria were age below 18 years, patients who were pregnant or lactating, haematocrit below 0.25, immune-modulating therapy except for low dose steroids, chemotherapy or radiation therapy within one year of inclusion, life-threatening bleeding, and ICU stay shorter than 4 days.

### Assessment of organ dysfunction and illness severity

The extent of organ dysfunction and illness severity were evaluated using the APACHE II score [17] at ICU admission and the sequential organ failure assessment (SOFA) score [18] daily during the observation period.

### Blood sampling

Arterial blood samples were drawn on days 1, 2, 3, and 4 of the ICU stay. Venous blood samples were used in the healthy control group to avoid complications related to arterial puncture.

### Isolation of PBMCs

PBMCs were isolated from heparinised blood using density gradient centrifugation [19] with lymphoprep 1.077 g/ml (Nycomed pharma, Norway). PBMCs were harvested from the interface and washed twice in RPMI-1640 supplemented with 2% foetal calf serum. The PBMCs were resuspended in foetal calf serum and freeze media (20% DMSO in RPMI-1640) and cryopreserved at - 80°C until analysis.

### Flow cytometry for the determination of viability and monocyte expression of CD163 and MR

The expression of monocyte-bound CD163 and MR and viability were determined by flow cytometry. The cells were thawed and washed in RPMI 1640 supplemented with 2% FCS. The cells were resuspended at a final concentration of 1×10^6^ PBMCs/ml. 100 μl of the PBMC suspension was stained with 7-AAD (BD Biosciences, San Jose, CA, USA) and leucogate (CD14PE/CD45FITC, BD Biosciences, San Jose, CA, USA) to determine viability. A separate aliquot of cells was stained with a cocktail of optimised quantities of antibodies against CD14 (anti-CD14E-PE-Cy7, clone M5E2, BD Biosciences, San Jose, CA, USA), CD16 (anti-CD16-APC-Cy7, clone 3G8 BD Biosciences, San Jose, CA, USA), CD163 (anti-CD163-APC, clone GHI/61 BioLegend, San Diego, CA, USA), and MR (anti-CD206-FITC, clone 19.2 BD Biosciences, San Jose, CA, USA). The cells were incubated in the dark for 15 min at room temperature. Following incubation, the cells were washed in 2 ml of cold PBS, pH 7.4, containing 0.5% bovine serum albumin and 0.1% Na-azide, followed by fixation in 250 μl of cold fixation buffer (PBS pH 7.4 containing 0.5% bovine serum albumin, 0.1% Na-azide, and 1% formaldehyde). The flow cytometric analysis was performed within 1 hour using a FACS Canto analyser (BD Biosciences, San Jose, CA, USA). Monocytes were identified based on their forward scatter and side scatter appearance and phenotypical defined as CD14 positive and CD45 positive. We recorded 30,000 events for each sample. In samples with low cell numbers, the sample was analysed for 300 seconds. CD14 was used as a marker of monocytes [20]. The gating for CD14 was based on isotype control antibodies. The monocyte expression of CD163 and MR was estimated by quantifying the median fluorescence intensity (MFI). The data were analysed with FlowJo software version 9.3.1 (Tree Star, Inc., Oregon, USA).

### Sandwich ELISA to determine the concentration of sCD163 and sMR

Samples for the determination of sCD163 and sMR were drawn in 9 ml EDTA tubes. The tubes were centrifuged at 4°C for 10 min at 3000 rpm. The plasma was then removed, aliquoted and stored at -80°C until analysis.

The levels of serum sCD163 and sMR were analysed in duplicate using samples of frozen serum with an in-house sandwich ELISA on a BEP2000 ELISA analyser (Dade Behring, Marburg, Germany) as previously described [21] (submitted to *Journal Of Leukocyte Biology*). Control samples were included in each run. The inter-assay imprecision in the current study for sCD163 was 1.9 CV% and 5.2 CV% at levels of 1.91 mg/l and 3.52 mg/l, respectively. The sMR imprecision was 3.5 CV% and 4.2 CV% at levels of 0.51 mg/l and 1.13 mg/l, respectively. Both sCD163 and sMR are resistant to thawing degradation, and their stability has been verified at −80°C for at least 12 months.

### Statistical analysis

Statistical differences between groups at inclusion were evaluated using a Wilcoxon-Mann-Whitney signed rank test. Differences between groups and development over time were evaluated using ANOVA repeated measurements with Greenhouse-Geisser correction. The data were log-transformed to ensure a normal distribution based on evaluation of qq-plots and histograms. Calculation of the area under the receiver operating characteristics (AUROC) curve was used to asses the diagnostic and prognostic values of each biomarker. Spearmańs rank correlation was used to examine correlations at admission. To examine correlations over time, multilevel mixed-effects linear regressions were applied. The patient inclusion number was used as a grouping variable and the model allowed for random slope and intercepts on biomarkers. A *p*-value < 0.05 was considered significant. Statistical analyses were performed using STATA 11.2 (StataCorp LP, College Station, Texas, USA).

## Results

### Patient characteristics

Patient characteristics are shown in Tables 1 and 2. There was no difference between the septic and non-septic patients with respect to the APACHE II score at inclusion and the SOFA score at inclusion or during the four-day observation period. The levels of CRP were higher in the septic patients than in the non-septic patients both at inclusion and during the four-day observation period. The septic and non-septic patients were similar with respect to the type of ventilation, and use of dialysis. The septic patients were more likely to receive inotropic agents, antibiotics and glucocorticoids than the non-septic patients in this study. The non-septic patients receiving antibiotics within the four-day observation period were either trauma patients receiving antibiotics prophylactic due to open fractures or patients with intracranial haemorrhage who developed a urinary tract infection during ICU stay.

**Table 2:** SOFA score and biochemical variables during the study period.

		Sepsis	Non-sepsis	Control
	day	*n* = 21	*n* = 15	*n* = 15
SOFA score	1	8 (7−12)	8 (6−11)	-
	2	10 (8−12)	9 (6−11)	-
	3	8 (5−11)	8 (4−10)	-
	4	8 (5−9)	7 (3−9)	-
WBC (x 10^9^/l)	1	11.55 (8.3−21.3)^1^^,^ 3	12.1 (10.5−14.1)^1^	5.1 (4.5−6.6)
	2	13.5 (9.63−21.2)	11.1 (8.1−12.9)	-
	3	14.3 (12.6−18.2)	9.7 (7.7−11.6)	-
	4	16.2 (12.6−18.7)	9.4 (6.5−10.9)	-
Monocytes (x 10^9^/l)	1	0.55 (0.23−0.98)^2^^,^ 4	1.08 (0.88−1.56)^1^	0.44 (0.38−0.57)
	2	0.62 (0.29−1.04)	1.12 (0.69−1.38)	-
	3	0.67 (0.39−1.02)	0.84 (0.66−1.5)	-
	4	0.77 (0.56−1.55)	0.84 (0.62−1.19)	-
CD163 (MFI)	1	799 (491−1568)^1^^,^ 2 ^,^ 3	433 (264−638)^1^	274 (213−340)
	2	1055 (294−1407)	297 (219−470)	-
	3	947 (649−1208)	219 (170−426)	-
	4	542 (300−820)	173 (85.8−269)	-
sCD163 (mg/ml)	1	3.70 (2.19−11−18)^1^^,^ 2 ^,^ 3	1.31 (0.94−1.48	1.35 (1.00−1.60)
	2	5.04 (2.09−11.21)	1.36 (1.00−1.61)	−
	3	4.98 (2.62−14.87)	1.34 (1.01−1.65)	−
	4	5.54 (2.95−14.27)	1.58 (1.08−1.88)	−
sMR (mg/ml)	1	1.11 (0.87−1.94)^1^^,^ 2 ^,^ 3	0.35 (0.29−0.46)^1^	0.24 (0.2−0.33)
	2	1.22 (0.98−1.98)	0.47 (0.31−0.55)	−
	3	1.39 (1.26−2.15)	0.51 (0.37−0.66)	−
	4	1.52 (1.38−2.29)	0.56 (0.35−0.71)	−
CRP (mg/l)	1	142.2 (109.6−277.5)^1^^,^ 2 ^,^ 3 ^,^ 4	47.15 (24.7−89)^1^	0 (0−0.7)
	2	229.7 (117.7−282.9)	88.1 (32.8−159.8)	−
	3	151.9 (111.1−283.1)	84.2 (33.1−138.5)	−
	4	74.4 (59.1−149.8)	65.2 (16.4−117.9)	−

SOFA Sequential Organ Failure Assessment score, WBC white blood cell, CRP C-reactive protein. Data are shown as the median with Inter-quartile range (IQR).

1 Significant compared with healthy controls (Wilcoxon signed rank-sum test).

2 Significant compared with non-septic patients (Wilcoxon signed rank-sum test).

3 Levels differ significantly between septic and non-septic patients during the observation period (two-way repeated measures ANOVA).

4 Significant time/group interaction between septic and non-septic patients during the observation period (two-way repeated measures ANOVA).

All septic patients had blood cultures drawn. The primary site of infection was the lungs (15/21), followed by the abdomen (5/21), and the urinary tract (1/21).

### White blood cell (WBC) count, monocyte count, distribution and viability

The WBC counts did not differ between septic and non-septic patients at inclusion but separated over time. Both groups had higher WBC counts than healthy controls.

At ICU admission, the monocyte count was significantly higher in the non-septic patients compared with the septic patients and the healthy controls. The septic patients and healthy controls had similar monocyte count, which were within normal range (Table 2). There was no difference in the CD45+, CD14+ monocyte fraction of total PBMCs between septic and non-septic patients. Both groups had a higher fraction of these cells than the healthy controls (data not shown). The viability within the monocyte gate was similar in all groups and above 90% in all samples (data not shown).

### Expression of monocyte-bound CD163 and levels of sCD163 at ICU admission and during the first four days of ICU stay

**Figure 1:**
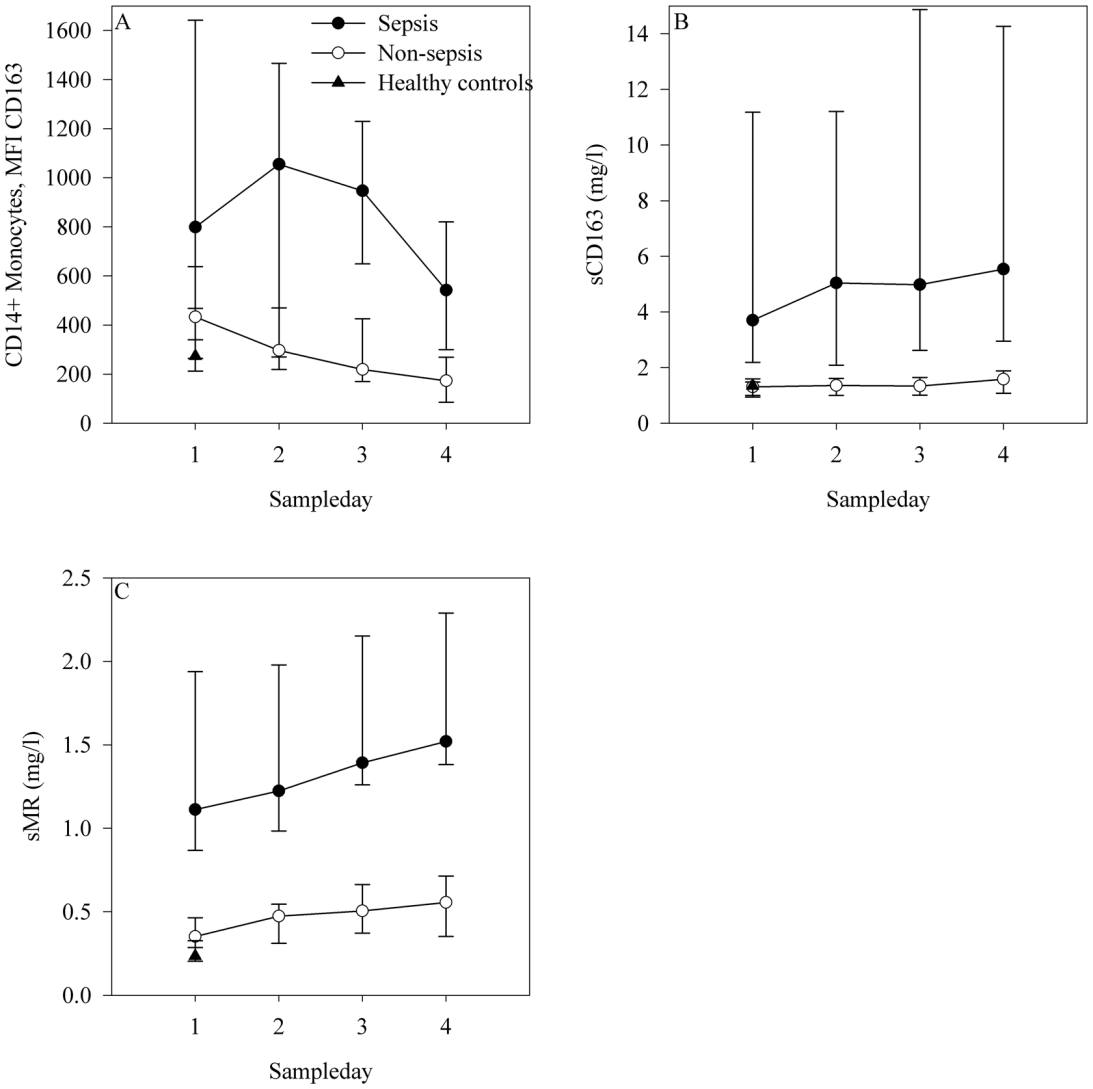
Levels of sCD163, sMR and the expression of monocyte-bound CD163 during the four-day observation period. A-C: At ICU admission and during the four-day observation period, expression of monocyte-bound CD163 (Panel A), sCD163 (Panel B), and sMR (Panel C) was significantly higher in septic patients compared with non-septic patients and healthy controls. The x-axis represents time and the y-axis represents the expression or level of each potential biomarker. Dots represent the median, bars represent the interquartile range.

At ICU admission, the monocyte expression of CD163 was higher in the septic patients compared with the non-septic patients (*p* = 0.01) and healthy controls (*p*<0.001). The monocyte expression of CD163 was higher in the non-septic patients compared with healthy controls (*p* = 0.01). During the four-day observation period, the monocyte expression of CD163 was significantly higher in the severe septic patients compared with non-septic patients (*p*<0.001) (Table 2 and Fig. 1A).

At ICU admission, the levels of sCD163 were higher in the septic patients compared with the non-septic patients and the healthy controls (*p*<0.001 for both comparisons). We observed no difference between the non-septic patients and the healthy controls. During the four-day observation period, the levels of sCD163 were significantly higher in the septic patients compared with the non-septic patients (*p*<0.001) (Table 2 and Fig. 1B).

There was no correlation between levels of sCD163 and expression of monocyte-bound CD163, neither at admission nor during the observation phase.

### Levels of sMR and expression of monocyte-bound MR at ICU admission and during the first four days of ICU stay

At ICU admission, the levels of sMR were significantly higher in the septic patients compared with the non-septic patients and the healthy controls (*p*<0.001 for both comparisons). The sMR level was also significantly higher in the non-septic patients compared with the healthy controls (*p* = 0.002). During the four-day observation period, the levels of sMR were significantly higher in the septic patients compared with the non-septic patients (*p*<0.001) (Table 2 and Fig. 1C).

We did not observe any expression of monocyte-bound MR.

### Abilities of sCD163, sMR, and monocyte-bound CD163 to discriminate between septic and non-septic patients

**Figure 2:**
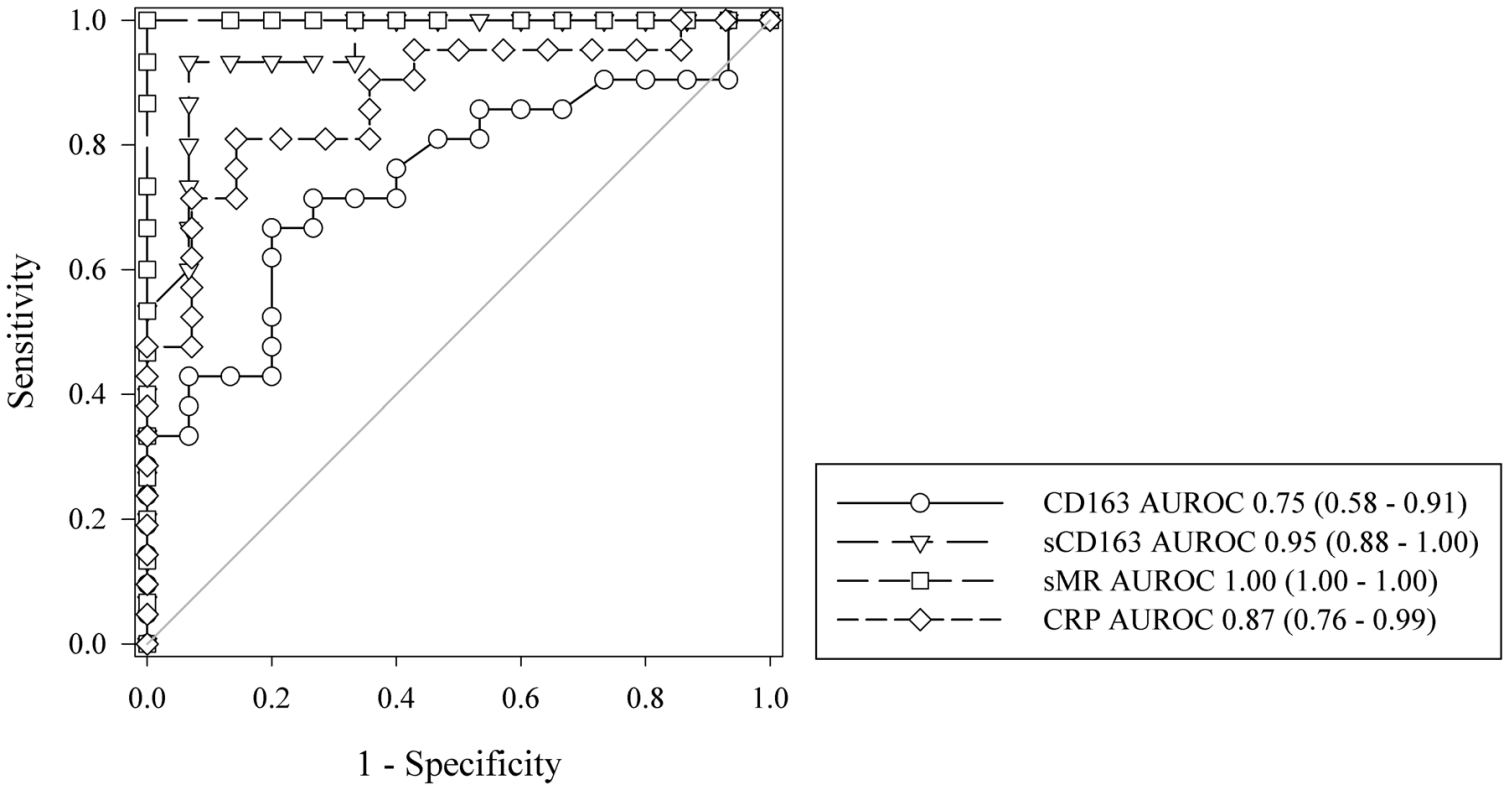
Receiver operating characteristic curve analysis and the ability to discriminate between septic patients and non-septic patients at ICU admission. Area under the receiver operating characteristic curve (AUROC) is shown for monocyte-bound CD163, sCD163, sMR, and plasma CRP. Numbers in parentheses are 95% confidence intervals.

To examine if the monocyte expression of CD163 and levels of sCD163 and sMR could be used to discriminate between septic patients and non-septic patients, we performed AUROC curve analysis. At ICU admission, sMR had the highest AUROC (1; 95% CI 1 to 1), followed by sCD163 (0.95; 95% CI 0.88 to 1) and monocyte-bound CD163 expression (0.75; 95% CI 0.58 to 0.91) (Fig. 2). There was no difference between sMR and sCD163, and both markers performed better than monocyte-bound CD163. The AUROC for sMR was significantly higher than the AUROC for plasma CRP (0.87, 95% CI 0.76 to 0.99) (*p* = 0.04). There was no difference in the AUROC between plasma CRP and monocyte-bound CD163 or between plasma CRP and sCD163.

sMR at a cut-off value of 0.61 mg/ml were able to discriminate between septic and non-septic patients with 100% sensitivity and 100% specificity. sCD163 at a cut-off value of 1.74 mg/ml had 93% sensitivity and 93% specificity. Monocyte-bound CD163 at a MFI cut-off value of 646.5 had 67% sensitivity and 80% specificity. CRP at a cut-off value of 91.8 mg/l had 80% sensitivity and 86% specificity.

### Expression of monocyte-bound CD163 and levels of sCD163 and sMR in in-hospital survivors and non-survivors within the septic group

**Figure 3:**
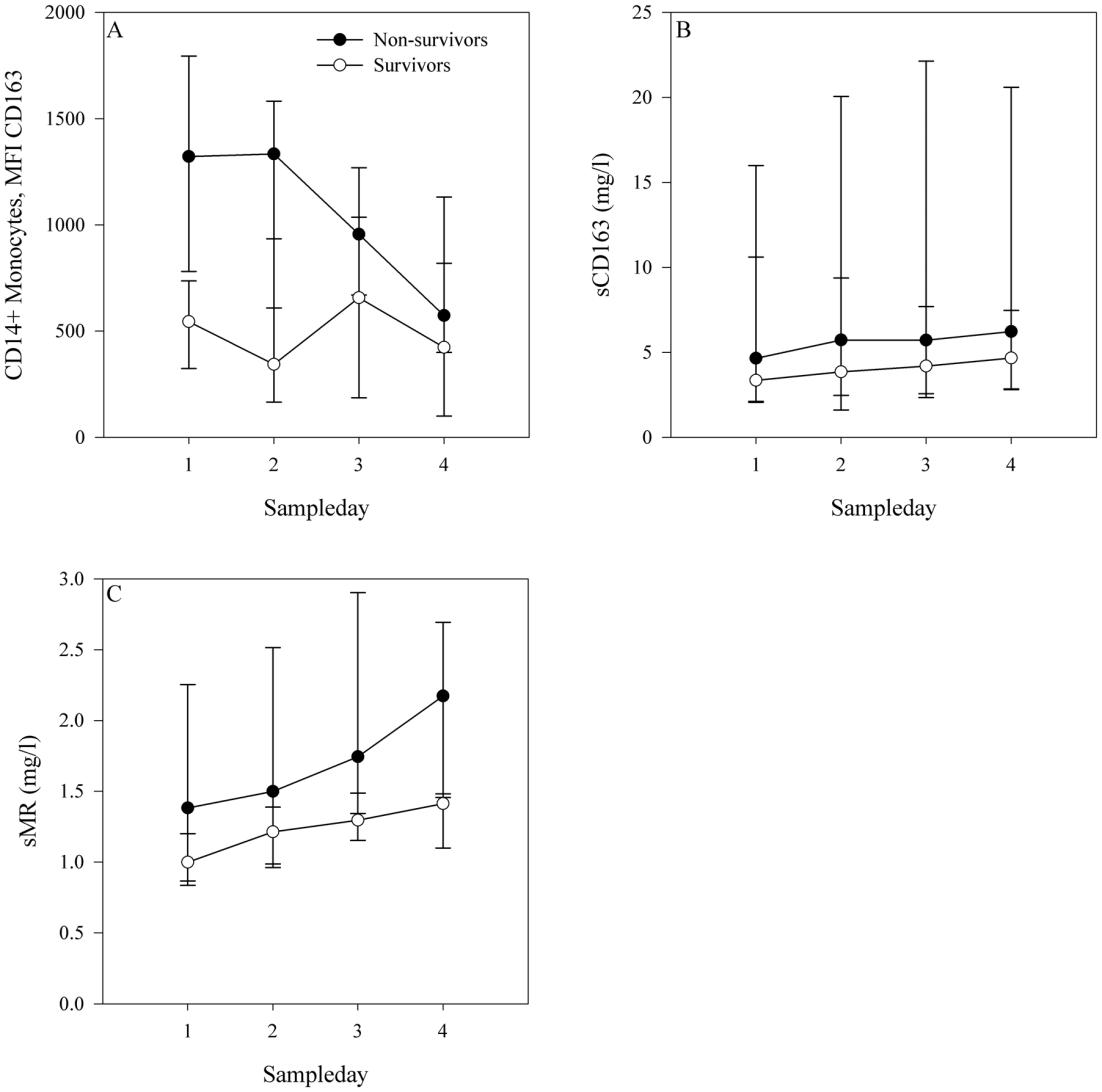
Levels of sCD163, sMR and the expression of monocyte-bound CD163 in in-hospital survivors and non-survivors within the septic group during the four-day observation period. A: Monocyte expression of CD163 was higher in non-survivors compared with survivors, both at ICU admission and during the four-day observation period. B + C: There were no difference in the levels of sCD163 (Panel B) or sMR (Panel C) between survivors and non-survivors, neither at ICU admission nor during the observation period. The x-axis represents time and the y-axis represents the expression or level of each potential biomarker. Dots represent the median, bars represent the interquartile range.

**Figure 4:**
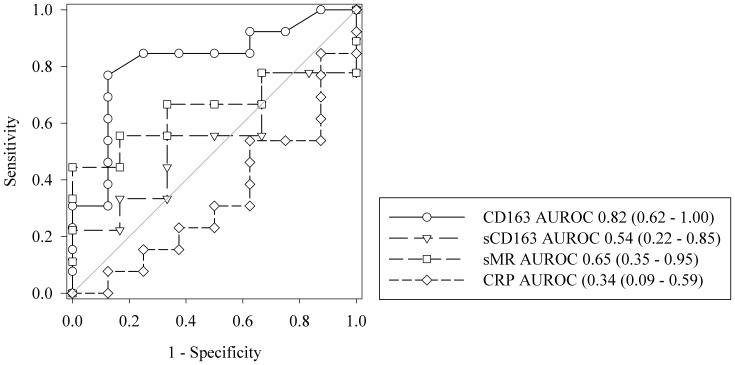
Receiver operating characteristic curve analysis and the ability to discriminate between survivors and non-survivors among the septic patients at ICU admission. Area under the receiver operating characteristic curve (AUROC) is shown for monocyte-bound CD163, sCD163, sMR, and plasma CRP. Numbers in parentheses are 95% confidence intervals.

The in-hospital mortality rate was 62% (13/21) in the septic patients and 7% (1/15) in the non-septic patients. All deaths after ICU admission occurred during the hospital stay (follow-up 90 days). Among the septic patients, the monocyte expression of CD163 was higher in the non-survivors compared with the survivors at ICU admission (*p* = 0.02) and during the four-day observation period (*p* = 0.006) (Fig. 3A). The AUROC at ICU admission for monocyte-bound CD163 ability to discriminate between survivors and non-survivors was 0.82; 95% CI 0.62 to 1 (Fig. 4). The monocyte expression of CD163 at a MFI cut-off value of 781.5 was able to discriminate between survivors and non-survivors with 77% sensitivity and 88% specificity.

We observed no difference in the levels of sCD163, sMR, and plasma CRP between survivors and non-survivors at ICU admission or during the four-day observation period (Fig. 3B+C). Thus sCD163, sMR, and CRP levels could not discriminate between survivors and non-survivors (Fig. 4).

## Discussion

The present work demonstrates that serum concentrations of the macrophage-related proteins sMR, sCD163 and monocyte-bound CD163 expression are higher in patients with severe sepsis or septic shock compared with critically ill non-septic patients.

The sMR was recently documented to be present in human serum [11], therefore little is known about how the serum concentration changes under pathological conditions. In our previous study, we found, in line with the present study, that the highest serum concentrations were in critically ill patients with sepsis, but also in patients with severe liver disease [11]. The present study is the first to investigate the levels of sMR in sepsis and its potential to discriminate between septic and non-septic patients. We found that the levels of sMR were higher in the septic patients compared with the non-septic patients and healthy controls and was able to discriminate between groups.

Soluble CD163 is a well-known marker of macrophage activation. Our findings of increased sCD163 levels in septic patients are in agreement with previous studies that reported 1.6 times higher concentration of sCD163 in critically ill patients compared with healthy controls at admission [13]. Additionally, a previous study has reported that the levels of sCD163 are superior to both CRP and procalcitonin in differentiating between septic and non-septic patients [14], this could not be demonstrated in the present study.

We also studied monocyte-bound CD163 expression and found that it was significantly increased in the septic patients compared with the non-septic patients, and thus able to discriminate between the two conditions, in accordance with previous results [22].

We did not observe any monocyte expression of MR, confirming the general belief that monocytes do not express MR [12]. However, recent studies have found weak monocyte expression of MR [22], [23].

Our overall aim in this study was to investigate whether levels of macrophage related proteins in their soluble and monocyte-bound form were able to discriminate between septic and non-septic patients. The monocyte expression of CD163, sCD163, and sMR as well as levels of CRP were able to discriminate between the groups. There was no difference between the ability of sMR and sCD163 to discriminate between the septic and the non-septic patients. Both soluble markers performed better than expression of monocyte-bound CD163. However, only sMR performed significantly better than plasma CRP in discriminating the septic patients from the non-septic patients. sMR was able to discriminate between septic patients and non-septic patients at ICU admission with an AUROC of 1.00, suggesting that sMR is an ideal biomarker for diagnosing sepsis. This suggests that specific macrophage-related biomarkers that reflect macrophage activation may indicate infectious inflammation in sepsis better than the non-specific CRP [15].

We also evaluated the ability of sMR, sCD163 and monocyte-bound CD163 to predict in-hospital mortality within the septic group. Monocyte-bound CD163 had the highest AUROC for the ability to discriminate between non-survivors and survivors at ICU admission, and was the only marker of the investigated macrophage-related markers that performed better than chance. Previous studies have reported a significant correlation between the levels of sCD163 at hospital admission or time of diagnosis and patient mortality. The correlation has been made in studies of patients with sepsis/bacteraemia [10], [14], [24] and in more general studies of ICU patients [13]. The reason that we are not able to reproduce this correlation in our study could be a lack of power due to the limited number of patients included.

Because circulating monocytes also express the investigated macrophage-related biomarkers, soluble levels of these biomarkers could potentially just reflect increased shedding from circulating monocytes instead of inflammation associated tissue macrophage activation. However, we did not observe any correlation between sCD163 and monocyte-bound CD163, suggesting that sCD163 does reflect tissue macrophage activation.

These preliminary results have to be taken cautiously and call for confirmation in a larger cohort. This is the first work on sMR in a well characterised group of septic patients, therefore the “perfect” power of sMR in identifying septic patients has to be interpreted with reservation. However, since our septic cohort are comparable to others in terms of organ dysfunction and illness severity and our results on sCD163 and surface bound CD163 are supported by other studies, we believe that our results on sMR indicates, that this soluble receptor has important characteristics and properties in sepsis. The most important limitation of the present study is the small sample size. The included 21 patients with severe sepsis or septic shock all required more than four days of ICU treatment making them *a priori* expected to have a high morbidity and mortality. Moreover, the requirement of at least four days of ICU treatment would generate a somewhat homogenous group of septic patients in contrast to the very heterogeneous nature of this large group of patients. The non-septic group consisted of patients with mono-organ failure in contrast to the septic patients who generally suffered from multi-organ failure. How this difference could affect our results remains to be clarified. Also of importance, the onset of disease is unknown in the septic group in contrast to the non-septic patients suffering from intracranial haemorrhage or trauma. This heterogeneity, in terms of time from onset of disease may blur our results but it also reflects the daily ICU setting.

## Conclusion

In conclusion, the present study supports the importance of macrophages in the sepsis pathogenesis in general and highlights the potential of macrophage-related proteins as biomarkers of sepsis. Especially, the potential future use of sMR as a sepsis biomarker is promising.
